# Maternal Allopurinol Prevents Cardiac Dysfunction in Adult Male Offspring Programmed by Chronic Hypoxia During Pregnancy

**DOI:** 10.1161/HYPERTENSIONAHA.118.11363

**Published:** 2018-08-27

**Authors:** Youguo Niu, Andrew D. Kane, Ciara M. Lusby, Beth J. Allison, Yi Yi Chua, Joepe J. Kaandorp, Rhiannon Nevin-Dolan, Thomas J. Ashmore, Heather L. Blackmore, Jan B. Derks, Susan E. Ozanne, Dino A. Giussani

**Affiliations:** 1 From the Department of Physiology, Development, and Neuroscience, University of Cambridge, United Kingdom (Y.N., A.D.K., C.M.L., B.J.A., Y.Y.C., R.N.-D., D.A.G.); 2 University Medical Center, Utrecht, the Netherlands (J.J.K., J.B.D.); 3 University of Cambridge Metabolic Research Laboratories and Medical Research Council Metabolic Diseases Unit, Institute of Metabolic Science, Addenbrooke’s Hospital, Cambridge, United Kingdom (T.J.A., H.L.B., S.E.O.); 4 Cambridge Cardiovascular Strategic Research Initiative (Y.N., S.E.O., D.A.G.).

**Keywords:** allopurinol, developmental programming, hypoxia, oxidative stress, pregnancy, rats

## Abstract

Integrating functional and molecular levels, we investigated the effects of maternal treatment with a xanthine oxidase inhibitor on the programming of cardiac dysfunction in adult offspring using an established rat model of hypoxic pregnancy. Female Wistar rats were divided into normoxic or hypoxic (13% O_2_) pregnancy±maternal allopurinol treatment (30 mg kg^-1^ d^-1^). At 4 months, hearts were isolated from 1 male per litter per outcome variable to determine cardiac function and responses to ischemia-reperfusion in a Langendorff preparation. Sympathetic dominance, perfusate CK (creatine kinase) and LDH (lactate dehydrogenase) and the cardiac protein expression of the β_1_-adrenergic receptor, the M_2_ Ach receptor (muscarinic type-2 acetylcholine receptor), and the SERCA2a (sarcoplasmic reticulum Ca^2+^ ATPase 2a) were determined. Relative to controls, offspring from hypoxic pregnancy showed elevated left ventricular end diastolic pressure (+34.7%), enhanced contractility (dP/dt_max_, +41.6%), reduced coronary flow rate (−21%) and an impaired recovery to ischemia-reperfusion (left ventricular diastolic pressure, area under the curve recovery −19.1%; all *P*<0.05). Increased sympathetic reactivity (heart rate, +755.5%; left ventricular diastolic pressure, +418.9%) contributed to the enhanced myocardial contractility (*P*<0.05). Perfusate CK (+431%) and LDH (+251.3%) and the cardiac expression of SERCA2a (+71.4%) were also elevated (*P*<0.05), further linking molecular markers of cardiac stress and injury to dysfunction. Maternal allopurinol restored all functional and molecular indices of cardiac pathology. The data support a link between xanthine oxidase–derived oxidative stress in hypoxic pregnancy and cardiac dysfunction in the adult offspring, providing a target for early intervention in the developmental programming of heart disease.

## Abstract 2

Supplemental Digital Content is available in the text.

Heart disease is a major health challenge worldwide, accounting for 1 in 3 deaths per year globally.^1–3^ Therefore, there is interest in identifying mechanisms underlying cardiovascular disease to design preventative strategies. It is established that traditional lifestyle risk factors, such as smoking, an unhealthy diet, obesity, and physical inactivity interact with our genes to set an increased risk of cardiovascular disease.^4^ It has also become established that the gene-environment interaction early in life may be just as, if not more, important in programming heart health and heart disease in the offspring.^5^ We, and others, have shown that chronic fetal hypoxia, the most common consequence of complicated pregnancy, can trigger a fetal origin of cardiac dysfunction and program an increased risk of heart disease in the adult offspring.^6–8^ Several studies in animal models have reported increased molecular markers of oxidative stress in cardiovascular tissues of fetal offspring of hypoxic pregnancy,^6–9^ and we reported that maternal treatment with the antioxidant vitamin C prevented the developmental programming of cardiovascular dysfunction in the adult offspring of hypoxic pregnancy in rats.^6,10^ Although the latter studies provide proof of principle that maternal antioxidant therapy may protect cardiac function in the adult offspring of complicated pregnancy, only high doses of vitamin C incompatible with human clinical translation proved effective.^6,10^

An alternative antioxidant strategy of improved translational value to human clinical therapy may be the xanthine oxidase inhibitor allopurinol. Hypoxia leads to an increase in xanthine oxidase–derived free radical generation,^11^ and in humans, maternal treatment with allopurinol crosses the placenta,^12^ justifying this route of administration for preventative therapy in obstetric practice. It has been suggested that allopurinol has beneficial effects in reducing ischemia-reperfusion (IR) damage in adult cardiology and in pediatric and adult cardiothoracic surgery.^13,14^ Indeed, maternal allopurinol treatment is currently being considered in human clinical trials to protect the newborn infant from oxidative stress–induced injury in pregnancy complicated by fetal hypoxia.^15^

Recently, we established a rat model in which maternal oral treatment with allopurinol yields circulating concentrations in the fetus similar to those reported in a human clinical context and suppresses xanthine oxidase activity in the maternal, placental, and fetal tissues.^16^ However, whether maternal oral treatment with this dosing regimen of allopurinol protects against programmed cardiac dysfunction in the adult offspring in hypoxic pregnancy is not known. Therefore, this study tested the hypothesis that maternal allopurinol treatment is protective against programmed cardiac dysfunction in adult male offspring of hypoxic pregnancy. This was tested using an established rat model by investigating the effect of hypoxic pregnancy with and without maternal allopurinol treatment on basal and stimulated cardiac function and on the cardiac response to IR in the adult male offspring using an isolated Langendorff preparation. To address mechanisms mediating changes in cardiac reactivity, cardiac responses to increasing doses of selective muscarinic and β_1_-adrenergic agonists were investigated, and alterations in the protein expression of the β_1_-adrenergic and the M_2_ Ach receptors (muscarinic type-2 acetylcholine receptors) were determined. To further link molecular mechanisms to cardiac dysfunction, perfusate concentrations of CK (creatinine kinase) and LDH (lactate dehydrogenase) and the cardiac expression of the SERCA2a (sarcoplasmic reticulum Ca^2+^ ATPase 2a), common markers of cardiac stress and injury, were also established.

## Methods

### Data, Materials, and Code Disclosure Statement

The data that support the findings of this study are available from the corresponding author on reasonable request.

### Ethical Approval

This research was approved under the Animals (Scientific Procedures) Act 1986 Amendment Regulations 2012 after ethical review by the University of Cambridge Animal Welfare and Ethical Review Board.

### Animals

Wistar rats (Charles River, Ltd, Margate, United Kingdom) were housed individually in ventilated cages under standard conditions with free access to food (Special Diet Services, United Kingdom) and water. After 10 days of acclimatization, virgin females (n=56, 10–12 weeks of age) were paired with fertile male rats (minimum 12 weeks of age). The presence of a copulatory plug was considered day 0 of pregnancy (term is ca. 21 days).

### Hypoxic Pregnancy and Maternal Allopurinol Treatment

On day 6 of pregnancy, rats were divided using a random number generator into 4 groups (n=12 per group): normoxic and hypoxic pregnancy, with or without maternal oral allopurinol treatment (30 mg kg^-1^ d^-1^ in vehicle jelly). Hypoxia exposure and allopurinol treatment was from day 6 to 20 of gestation. The jelly containing allopurinol and the vehicle jelly were undistinguishable, but they were coded. Experimenters assigning treatment and analysing the data were unaware of the meaning of the code until all experiments and data analyses were completed. Scientists were, therefore, blinded to the group allocation. We have established that this maternal allopurinol treatment in rat pregnancy crosses the placenta, leads to therapeutic concentrations of allopurinol and its active metabolite oxypurinol in the fetal circulation, and suppresses xanthine oxidase activity in the mother, placenta, and fetus.^16,17^ Pregnant rats subjected to hypoxia were placed inside a transparent chamber, which received air and nitrogen to required mixture.^6^ The chamber could house 9 rat cages in a tranquil environment.^6^ Pregnancies undergoing hypoxia were maintained at 13% O_2_ from day 6 to 20 of gestation. This level of hypoxia equates to ≈3600 m, the altitude at which pregnancy complications significantly increase in human populations.^18,19^ This level of maternal hypoxia also leads to levels of fetal hypoxia associated with complications in sea level human pregnancy.^20,21^ We have previously reported that this level and duration of maternal hypoxia does not lead to alternations in maternal food intake.^6^ The onset of hypoxia was on day 6, as preliminary studies revealed, markedly enhanced pregnancy loss if the hypoxia started earlier.^6^ At birth, each pup within each litter was sexed by measurement of anogenital distance and pups were weighed and culled to 8 per litter to standardize feeding and maternal care.

### Langendorff Heart Preparation

All pups remained with their mothers until weaning at postnatal day 21. After weaning, pups were group-housed under standard conditions and maintained until 4 months of age. At 4 months, to control for sex and within-litter variation, only 1 male pup per litter was used for each outcome. Unused offspring were donated to other studies. Adult males underwent euthanasia by CO_2_ inhalation and posterior cervical dislocation. The heart was rapidly excised, immediately placed in ice-cold Krebs-Henseleit bicarbonate buffer, and then mounted onto a Langendorff preparation, used routinely in our laboratory.^22^ In brief, a recirculating Krebs-Henseleit bicarbonate buffer solution containing (mmol/L) 120 NaCl, 4.7 KCl, 1.2 MgSO_2_.7H_2_O, 1.2 KH_2_PO_4_, 25 NaHCO_3_, 10 glucose, and 1.3 CaCl_2_.2H_2_O was filtered through a 5 μm cellulose nitrate filter (Millipore, Bedford, MA) and gassed with O_2_:CO_2_ (95:5) at 37°C. Isolated hearts were perfused in retrograde fashion through the aorta at a constant pressure (100 cm H_2_O). A small flexible nonelastic balloon was inserted into the left ventricle (LV) through the left atrium. The balloon was filled with distilled water, and its volume was adjusted to 150 μl to obtain an LV end-diastolic pressure (LVEDP) recording between 5 and 10 mm Hg.^22^ After 15-minute stabilization, basal heart rate, LV systolic pressure, and LVEDP were recorded. Basal LV developed pressure (LVDP) was calculated as LV systolic pressure–LVEDP. The maximum and minimum first derivatives of the LV pressure (dP/dt_max_ and dP/dt_min_) were calculated. Coronary flow rate was calculated by timed collections of perfusate.^22^

### Cardiac Stimulated Function: Agonists and IR Challenge

The cardiac responsiveness to the muscarinic agonist Carbachol (carbamylcholine chloride, Sigma-Aldrich, Co, Ltd, Poole, United Kingdom; range, 10^-10^–10^-6^mol/L) and the β_1_-adrenoreceptor agonist isoprenaline ([−]-isoprenaline [+]-bitartrate salt, Sigma-Aldrich, Co, Ltd, Poole, United Kingdom; range, 10^–11^–10^-7^mol/L) were investigated as described before.^22^ After the final dose response, when heart rate and LVDP recovered back to the baseline, the hearts were subjected to an IR challenge composed of 10 minutes of global ischemia by stopping the perfusion, followed by 30 minutes of reperfusion.

### CK and LDH Activity

The coronary effluent (2 mL) was collected before ischemia (−15 minutes) and at the onset of reperfusion (0 minutes). Samples were immediately frozen in liquid nitrogen and kept at −80°C until analysis. Perfusate CK activity was analyzed using a bichromatic coupled enzyme reaction assay (Siemens Dimension RxL analyser, Malvern, PA). Perfusate LDH activity was analysed using a colorimetric assay (Siemens Dimension RxL analyser, Malvern, PA).

### Cardiac Protein Expression

At 4 months of age, the heart from a male sibling from each litter was isolated, frozen in liquid nitrogen, and kept at −80°C. Protein expression of the cardiac β_1_-adrenergic receptor, the M_2_ Ach receptor, and the SERCA2a was determined by Western blot;^23^ 10 µg of protein were loaded for each sample. Equal protein loading was confirmed by Coomassie blue staining. Antibodies used were β_1_-adrenergic receptor (Santa Cruz Biotechnology, Dallas, TX), M_2_ Ach receptor (Abcam, Cambridge, United Kingdom), and the cardiac SERCA2a, Cell Signaling, Danvers, MA). Horseradish peroxidase-linked second antibody (Jackson ImmunoResearch) binding was detected using Super15 Signal West Pico Chemiluminescent substrate (Thermo Scientific, United Kingdom). Autoradiographed images were quantified using AlphaEase (Alpha Innotech).

### Statistical Analysis

Appropriate power calculations derived from previous data sets were performed to determine the minimum sample size required to achieve statistical significance set at *P*<0.05. Data are expressed as mean±SEM. Data were compared statistically by 2-way ANOVA, with hypoxia and allopurinol treatment as 2 between-subjects factors. The Tukey post hoc test was used to isolate significant differences.

## Results

### Basal Cardiac Function

**Figure 1.**
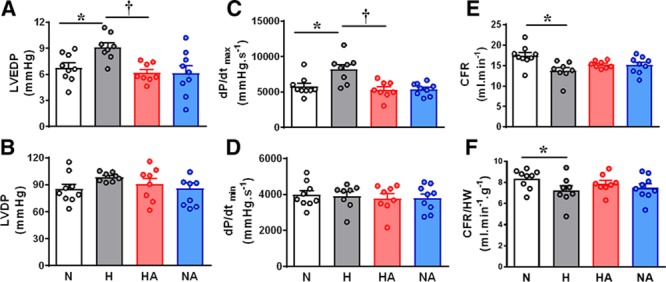
Cardiac basal function. Values are dot plots, as well as mean±SEM. Groups are normoxic (N; n=9), hypoxic (H; n=8), hypoxic treated with allopurinol (HA; n=8), and normoxic treated with allopurinol (NA; n=9) pregnancy. Significant differences (*P*<0.05) are *vs N; †vs H (2-way ANOVA+Tukey test). CFR indicates coronary flow rate; dP/dt _max_ and dP/dt _min_, the maximum and minimum first derivatives of the left ventricular pressure; HW, heart weight; LVDP, left ventricular developed pressure; and LVEDP, left ventricular end diastolic pressure.

Adult male offspring of hypoxic pregnancy showed elevated LVEDP with no change in LVDP (Figure 1A and 1B), a significant increase in dP/dt _max_ with no change in dP/dt _min_ (Figure 1C and 1D), and impaired coronary flow rate (Figure 1E and 1F). Maternal allopurinol prevented the increase in LVEDP and in dP/dt_max_ and the fall in coronary flow rate (Figure 1A, 1C, 1E, and 1F). Maternal allopurinol treatment in normoxic pregnancy did not affect basal function in hearts of adult male offspring (Figure 1A through 1F).

### Cardiac Responses to Autonomic Agonists

**Figure 2.**
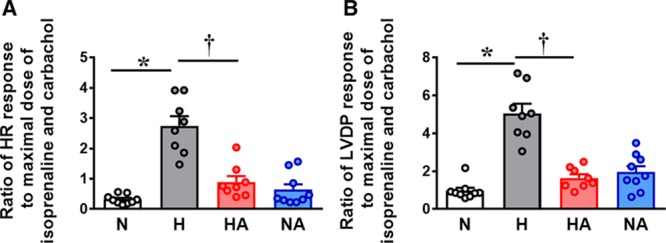
Cardiac sympathetic dominance. Values are dot plots, as well as mean±SEM for the ratio of cardiac chronotropic and inotropic responses to isoprenaline and carbachol. Groups are normoxic (N; n=9), hypoxic (H; n=8), hypoxic treated with allopurinol (HA; n=8), and normoxic treated with allopurinol (NA; n=9) pregnancy. Significant differences (*P*<0.05) are *vs N; †vs H (2-way ANOVA+Tukey test). HR indicates heart rate; and LVDP, left ventricular developed pressure.

Hearts of adult male offspring of normoxic pregnancy showed dose-dependent chronotropic (heart rate) and inotropic (LVDP) responses to agonists (Table S1 in the online-only Data Supplement). Adult male offspring of hypoxic pregnancy showed diminished cardiac negative chronotropic and inotropic responses to carbachol, whereas significantly enhanced positive chronotropic and inotropic responses to isoprenaline (Table S1). Consequently, the ratio of the maximal responses to Isoprenaline relative to those of carbachol was markedly increased (Figure 2). Hearts of adult male offspring of hypoxic pregnancy treated with allopurinol no longer showed these effects (Table S1 and Figure 2). Hearts of adult male offspring of normoxic pregnancy treated with allopurinol showed an impaired inotropic response to carbachol (Table S1).

### Ischemia/Reperfusion Challenge

**Figure 3.**
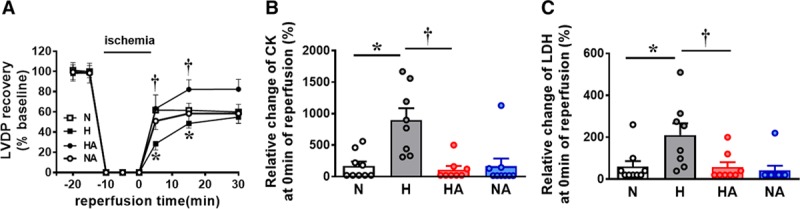
Cardiac ischemia-reperfusion challenge. Values are dot plots or mean±SEM for the cardiac left ventricular developed pressure (LVDP) response to ischemia-reperfusion and the perfusate CK (creatine kinase) and LDH (lactate dehydrogenase) levels. Groups are normoxic (N; n=9), hypoxic (H; n=8), hypoxic treated with allopurinol (HA; n=8), and normoxic treated with allopurinol (NA; n=9) pregnancy. Significant differences (*P*<0.05) are *vs N; †vs H (2-way ANOVA+Tukey test).

Adult male offspring of hypoxic pregnancy showed an impaired cardiac recovery to IR (Figure 3A) and enhanced CK and LDH effluent levels (Figure 3B and 3C) at the onset of reperfusion. Adult male offspring of hypoxic pregnancy treated with allopurinol showed a restored cardiac recovery response to IR and normalized CK and LDH (Figure 3A through 3C).

### Cardiac Protein Expression

**Figure 4.**
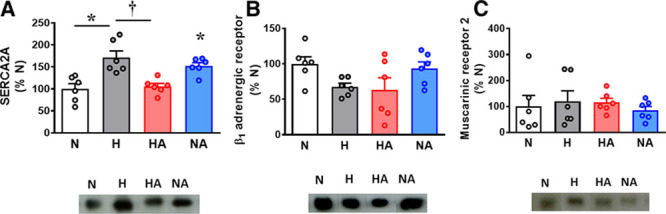
Cardiac proteins expression. Values are dot plots, as well as mean±SEM. Groups are normoxic (N; n=6), hypoxic (H; n=6), hypoxic treated with allopurinol (HA; n=6), and normoxic treated with allopurinol (NA; n=6) pregnancy. Significant differences (*P*<0.05) are *vs N; †vs H (2-way ANOVA+Tukey test). SERCA2A indicates sarcoplasmic reticulum Ca^2+^ ATPase 2a.

Adult male offspring of hypoxic pregnancy showed an increased cardiac protein expression of SERCA2a without any significant effects on β_1_-adrenergic or muscarinic type-2 receptor expression (Figure 4A through 4C). Adult male offspring of hypoxic pregnancy treated with allopurinol showed restored cardiac protein expression of SERCA2a (Figure 4A). Adult male offspring of normoxic pregnancy treated with allopurinol showed an enhanced cardiac protein expression of SERCA2a relative to hearts of adult male offspring of untreated normoxic pregnancy (Figure 4A) with no effects on the protein expression of β_1_-adrenergic or muscarinic type-2 receptors.

### Pregnancy Characteristics and Offspring Biometry

Values for gestation length, litter size and for the male to female ratio were not different between groups (Table). Hypoxic pregnancy did not affect birth weight or the symmetry of body growth. Relative to normoxic pregnancy, hearts of adult male offspring of hypoxic pregnancy were of similar weight, but the absolute weight of the left ventricle was reduced (Table). Maternal allopurinol in hypoxic pregnancy did not affect the pregnancy characteristics or the male offspring biometry, and it did not restore the reduction in absolute LV weight measured in adult male offspring of untreated hypoxic pregnancy (Table). Maternal treatment with allopurinol in normoxic pregnancy did not have any effects (Table).

**Table.**
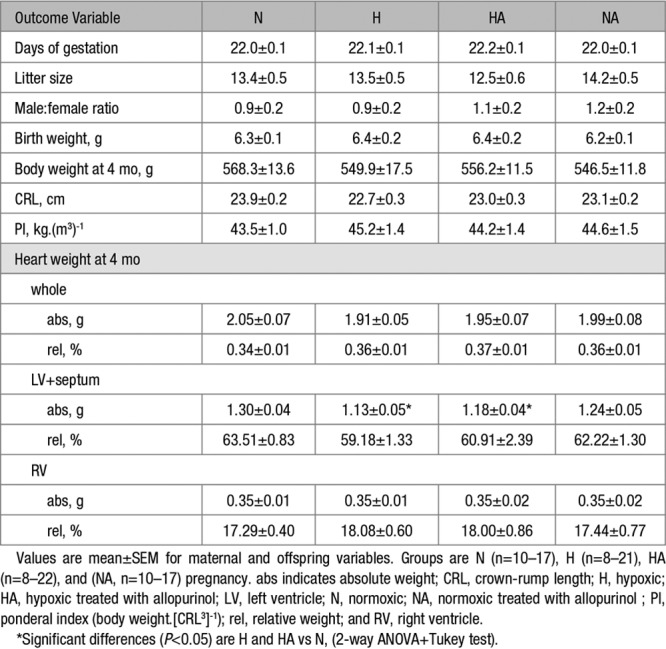
Pregnancy Characteristics and Offspring Biometry

## Discussion

**Figure 5.**
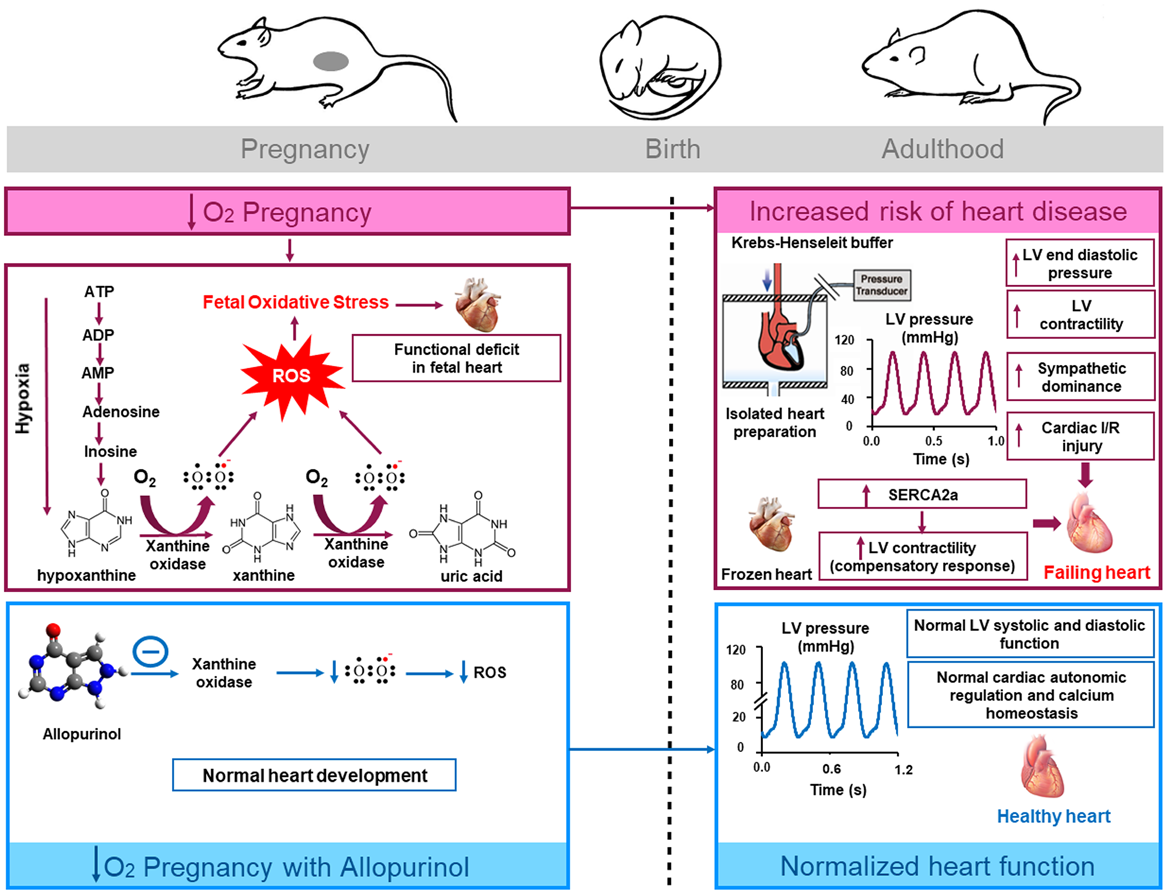
Summary of key findings. Hypoxic pregnancy programmes cardiac dysfunction in adult male offspring. Mechanisms involved include an increase in xanthine oxidase, cardiac sympathetic dominance, and altered cardiac calcium biology. Maternal treatment with the xanthine oxidase inhibitor, allopurinol, prevents the programmed cardiac dysfunction in adult male offspring, normalizing the cardiac sympathetic dominance and altered cardiac calcium biology. Therefore, oxidative stress derived from xanthine oxidase plays an important role in the programming of cardiac dysfunction by developmental hypoxia. Maternal treatment with allopurinol should be considered as clinical intervention against the programming of heart disease in adult offspring of human high-risk pregnancy. IR indicates ischemia-reperfusion; LV, left ventricle; ROS, reactive oxygen species; and SERCA2a, sarcoplasmic reticulum Ca2+ ATPase 2a.

We show that maternal treatment with allopurinol using a dose regimen that inhibits xanthine oxidase activation in the maternal, placental, and fetal tissues restores all functional and molecular markers of cardiac pathology in adult male offspring of hypoxic pregnancy. Mechanisms contributing to the enhanced cardiac contractility in hearts of adult male offspring of hypoxic pregnancy include a programmed increase in cardiac sympathetic dominance. This is characterized by reciprocal effects on the cardiac responsiveness to β_1_-adrenergic and muscarinic receptor stimulation, without affecting their receptor protein expression. Mechanisms contributing to alterations in basal cardiac function in adult male offspring of hypoxic pregnancy also include an increase in the protein expression of SERCA2a, indicating a reorganization of cardiac Ca^2+^ management. The activities of CK and LDH in the coronary perfusate after IR in adult male offspring of hypoxic pregnancy were also elevated, thereby, linking molecular markers of cardiac stress and injury to the increased cardiac susceptibility to IR injury (Figure 5).

Elevated LVEDP in the present study suggests impaired LV relaxation during diastole, indicative of cardiac diastolic dysfunction and increased cardiac stiffness, both of which are associated with increased mortality resulting from a cardiac event.^24^ In an elegant study, Rueda-Clausen et al^8^ also reported echocardiographic evidence of elevated LVEDP without significant effects on LVDP in adult rat offspring of hypoxic pregnancy. Elevated LVEDP is an important index because, clinically, cardiac diastolic dysfunction often precedes systolic dysfunction.^25^ The elevated diastolic pressure in hearts of male offspring of hypoxic pregnancy is likely because of increased diastolic Ca^2+^. The increase in cardiac SERCA upregulation also evident in the male offspring of hypoxic pregnancy may, therefore, be a compensatory mechanism to offset the increase in diastolic Ca^2+^. The mechanism underlying the increase in diastolic Ca^2+^ includes an enhanced RyR (ryanodine receptor) leak, also known to be exacerbated by the upregulation of SERCA, which will increase sarcoplasmic reticulum Ca^2+^ load and thereby release.^26,27^ In turn, an increase in sarcoplasmic reticulum Ca^2+^ release will lead to greater contractility by increasing the amplitude of the Ca^2+^ transient. The latter is consistent with the significantly greater increase in dP/dt_max_ in hearts of offspring of hypoxic pregnancy in the present study.

Additional data in the present study show increased sympathetic dominance in hearts of male offspring of hypoxic pregnancy. The increase in cardiac protein levels of SERCA2a in offspring from hypoxic pregnancy may again be involved in this programmed response, as SERCA2a promotes an increase in cardiac contractility and stimulation of cardiac β-adrenergic receptors phosphorylates and inactivates phospholamban, an inhibitor of SERCA.^28^ Increased cardiac sympathetic dominance is part of a compensatory neuroendocrine mechanism to increase myocardial contractility and maintain cardiac output in the young adult offspring of hypoxic pregnancy. However, such compensatory responses may become decompensated later in life, as sustained increases in sympathetic dominance and myocardial contractility are unsustainable, triggering deleterious effects on cardiac function and leading eventually to heart failure.^29,30^

A reduction in basal coronary flow rate has been linked with an increased risk of cardiac injury in response to IR.^30,31^ Consistent with this statement, hearts of adult male offspring of hypoxic pregnancy had an impaired IR recovery and enhanced LDH and CK in the coronary effluent after ischemia. LDH is involved in glucose metabolism and catalyzes the interconversion of lactate and pyruvate, whereas CK catalyses the conversion of creatine to phosphocreatine, responsible for ATP transfer and utilization. When cardiomyocytes are damaged, LDH and CK are released from the cytoplasm into the bloodstream, an effect that has been exploited clinically as established biomarkers for cardiac injury.^7,31^ An increase in the effluent levels of LDH and CK immediately after ischemia is, therefore, strong evidence for an increased risk of IR injury in hearts of offspring of hypoxic pregnancy.

We, and others, have previously reported that pregnancy complicated by chronic hypoxia programmes cardiac dysfunction in the adult offspring secondary to the generation of oxidative stress in the fetal cardiovascular system.^6–9^ By adulthood, levels of oxidative stress in the cardiovascular system are no longer different between groups. However, present data show that hypoxic pregnancy sets a functional deficit in the hearts of male offspring even by early adulthood with evidence of diastolic dysfunction, sympathetic dominance, and increased myocardial susceptibility to IR injury. Data in the present study show that xanthine oxidase–derived oxidative stress contributes to the programming of this cardiac dysfunction in adult male offspring of hypoxic pregnancy. Xanthine oxidase and its precursor, XDH (xanthine dehydrogenase), both catalyze the conversion of hypoxanthine to xanthine and xanthine to uric acid. They differ in that XDH utilises NAD^+^ (oxidized nicotinamide adenine dinucleotide) preferentially as the electron acceptor, whereas xanthine oxidase only reduces oxygen, resulting in the concomitant generation of the superoxide anion and hydrogen peroxide.^32^ Xanthine oxidase becomes a powerful pro-oxidant mechanism stimulated by chronic hypoxia, primarily because of facilitated conversion of XDH to xanthine oxidase and the accumulation of the substrate hypoxanthine.^33^ The gene expression of xanthine oxidase and XDH is also regulated by oxygen tension, which is activated by hypoxia and inhibited by hyperoxia.^34^ Therefore, maternal treatment with allopurinol by inhibiting xanthine oxidase activity in the fetoplacental unit may offer therapeutic potential to prevent the programming of cardiac dysfunction in adult offspring of pregnancies complicated by hypoxia. Indeed, increased sympathetic dominance in hearts of adult offspring seems to be a common programming mechanism in several types of adverse pregnancy, for instance, in maternal obesity during pregnancy.^23^ Therefore, maternal allopurinol may protect against cardiovascular dysfunction in the adult offspring programmed by several types of adverse pregnancy. In the present study, it is of additional interest that hearts of adult offspring from hypoxic pregnancies treated with allopurinol appeared to recover better from a period of IR, in terms of LVDP, compared with hearts from untreated and treated control pregnancies. This may be because of additional cardioprotective effects of allopurinol. For instance, evidence from animal models and human clinical studies report that in addition to its antioxidant properties, allopurinol enhances cardiac energy and mechanical efficiency by decreasing myocardial oxygen consumption, particularly in the failing heart.^35,36^ Maternal allopurinol treatment is currently being considered in human clinical trials to protect the newborn infant from oxidative stress–induced injury in pregnancy complicated by fetal hypoxia.^15^ Here, we show that maternal allopurinol transcends beneficial effects on the newborn infant to also protect against the programming of cardiac dysfunction in the male offspring. These findings are of significant human clinical importance as offspring of complicated pregnancy, despite the absence of intrauterine growth restriction and reduced birth weight, have been reported to already show cardiac dysfunction as children.^37^ A component of this cardiac dysfunction in children programmed by adverse intrauterine conditions may thus involve xanthine oxidase activation.

This study has limitations. Only male offspring were investigated to control for sex differences but not to address them. Maternal treatment with allopurinol in normoxic pregnancy also led to an increase in the cardiac protein levels of SERCA2a in the adult male offspring. The mechanism underlying this effect is not clear and further studies are required to address this. However, this effect was not matched by an increase in LVEDP or sympathetic dominance in hearts of adult male offspring of normoxic pregnancy treated with allopurinol. Therefore, maternal treatment with allopurinol should only be given to those who need it; that is in pregnancy diagnosed with chronic fetal hypoxia rather than to all pregnancies. This may not be feasible if risk cannot be identified before administration. Current obstetric opinion is that, in human pregnancy, chronic fetal hypoxia can be reliably diagnosed by ultrasound between 20 and 26 weeks of gestation, according to severity. Diagnosis encompasses early onset fetal growth restriction with reduced fetal heart rate variability and abnormal Doppler blood flow velocimetry indices, such as in those human pregnancies recently recruited to the EVERREST (Developing a Therapy for Fetal Growth Restriction) or TRUFFLE (Trial of Umbilical and Foetal Flow in Europe) studies.^38,39^ However, it must be highlighted that all these measurable outcomes are surrogate indices associated with rather than direct measures of chronic fetal hypoxia. There is, therefore, a need for further study and for clinical discussion to address these important limitations before translation.

## Perspectives

Developmental hypoxia, the most common outcome in human adverse pregnancy programmes an increased risk of heart disease in the adult offspring. Using an established rodent model, here, we show that xanthine oxidase–derived oxidative stress links developmental hypoxia with cardiac dysfunction and with molecular mechanisms of cardiac stress and injury in the adult offspring. In addition, maternal treatment with allopurinol is protective. Therefore, targeted inhibition of xanthine oxidase–derived oxidative stress may offer improved translational value to human clinical therapy to prevent the programming of cardiac dysfunction in offspring of high-risk pregnancy.

## Sources of Funding

This study was supported by the British Heart Foundation, London, United Kingdom. D. Giussani is the Professor of Developmental Cardiovascular Physiology and Medicine at the Department of Physiology, Development and Neuroscience at the University of Cambridge, Professorial Fellow and Director of Studies in Medicine at Gonville and Caius College, a Lister Institute Fellow, and a Royal Society Wolfson Research Merit Award Holder.

## Disclosures

None.

## Supplementary Material

## Novelty and Significance

### What Is New?

- Chronic fetal hypoxia, the most common complication in adverse pregnancy in humans, programmes cardiovascular dysfunction in the adult offspring. However, few studies have addressed mechanism and thereby intervention.
- We show that xanthine oxidase–derived oxidative stress links hypoxic pregnancy with programmed cardiovascular dysfunction in the adult offspring and that maternal treatment with allopurinol is protective.

### What Is Relevant?

- It is now accepted that the gene-environment interaction early in life may be just as, if not more, important in programming cardiovascular health and disease, including hypertension.
- Therefore, when considering strategies to reduce the burden of cardiovascular disease, a greater focus on prevention rather than treatment is needed and for candidate interventions to start as early as possible during the developmental trajectory rather than to wait until disease is established and irreversible.

### Summary

In this translational science study, we show that maternal treatment with allopurinol in adverse pregnancy protects against increased risk of heart disease in the adult offspring.
