# Supplementary material for: Maternal Allopurinol Prevents Cardiac Dysfunction in Adult Male Offspring Programmed by Chronic Hypoxia During Pregnancy
Source: Hypertension. 2018 Aug 27;72(4):971–8. doi: 10.1161/HYPERTENSIONAHA.118.11363 (PMC6135482; doi:10.1161/HYPERTENSIONAHA.118.11363)
Supplement: Supplementary file 1 [file hyp-72-971-s001.docx]

**Maternal Allopurinol Prevents Cardiac Dysfunction in Adult Male Offspring Programmed by Chronic Hypoxia during Pregnancy***

Youguo Niu, MD, D.Phil,^a,d^  Andrew D. Kane, MD, PhD,^a^  Ciara M. Lusby, M.Phil,^a^ Beth J. Allison, PhD,^a^ Yi Yi Chua, BA,^a^ Joepe J. Kaandorp, MD,^b^ Rhiannon Nevin-Dolan, BSc,^a^  Thomas J. Ashmore BSc,^c^ Heather L. Blackmore PhD,^c^  Jan B. Derks, MD, PhD,^b^ Susan E. Ozanne, PhD,^c,d^ Dino A. Giussani, PhD, ScD ^a,d^

^a^ Department of Physiology, Development and Neuroscience, University of Cambridge, UK;

^b^ University Medical Center, Utrecht, The Netherlands;

^c^ University of Cambridge Metabolic Research Laboratories and MRC Metabolic Diseases Unit, Institute of Metabolic Science, Addenbrooke's Hospital, Cambridge, UK;

^d^ Cambridge Cardiovascular Strategic Research Initiative.

* Part of this work has been awarded the Pfizer President’s Presenter’s Award at the 59th Annual Meeting of the Society for Reproductive Investigation, San Diego,USA, March, 2012

**Short Title**: Allopurinol and Programmed Cardiac Dysfunction

**Corresponding Author**:

Professor Dino A. Giussani PhD ScD FRCOG

Department of Physiology, Development and Neuroscience

University of Cambridge

Downing Street, Cambridge

CB2 3EG

UK

Email: [dag26@cam.ac.uk](mailto:dag26@cam.ac.uk)

Tel: +44-(0)1223 333894

Fax: +44-(0)1223 333840

**TABLE S1.** **Chronotropic and Inotropic Responses to Carbachol and Isoprenaline**

| Cardiac response | N | H | HA | NA |
| --- | --- | --- | --- | --- |
| HR response |  |  |  |  |
| Carbachol (M) |  |  |  |  |
| 10^-10^ | -3.35 ± 0.46 | -2.24± 0.39 | -4.07 ± 1.01 | -2.54 ± 0.83 |
| 10^-8^ | -9.82 ± 2.53 | -3.57 ± 0.79* | -10.46 ± 3.10 | -7.40 ± 1.72 |
| 10^-6^ | -53.88 ± 3.26 | -18.60± 2.90* | -29.82 ± 8.59* | -40.44 ± 6.01 |
| Isoprenaline (M) |  |  |  |  |
| 10^-11^ | 1.54 ± 0.21 | 1.66 ± 0.49 | 0.96 ± 0.66 | 2.53 ± 0.67 |
| 10^-9^ | 3.54 ± 1.11 | 8.17 ± 3.43 | 4.82± 1.21 | 5.81 ± 1.09 |
| 10^-7^ | 16.74 ± 2.32 | 46.16 ± 5.51* | 18.11 ± 3.30 | 16.49 ± 2.00 |
| LVDP response |  |  |  |  |
| Carbachol (M) |  |  |  |  |
| 10^-10^ | -6.83 ± 1.16 | -5.79 ± 0.95 | -12.90 ± 3.94 | -6.66 ± 0.74 |
| 10^-8^ | -17.36 ± 4.71 | -5.92 ± 0.93* | -13.86 ± 5.53 | -14.23 ± 2.19 |
| 10^-6^ | -65.76 ± 2.53 | -23.72 ± 3.18* | -41.74 ± 4.42*† | -51.20 ± 3.99*† |
| Isoprenaline (M) |  |  |  |  |
| 10^-11^ | 4.59 ± 0.72 | 8.48 ± 2.77 | 4.66 ± 0.76 | 5.22 ± 1.14 |
| 10^-9^ | 10.18 ± 2.40 | 27.28 ± 6.27* | 9.07 ± 1.50 | 12.01 ± 1.11 |
| 10^-7^ | 63.11 ± 9.29 | 157.67 ± 27.86* | 62.88 ± 2.95 | 98.98 ± 16.89 |

Values are mean ± S.E.M of percentage changes relative to baseline for heart rate and LVDP in hearts isolated from adult offspring. Groups are normoxic (N, n=9), hypoxic (H, n=8), hypoxic treated with Allopurinol (HA, n=8) and normoxic treated with Allopurinol (NA, n=9) pregnancy. Significant differences (P<0.05) are: *, *vs.* N; †, *vs.* H, (two way ANOVA+Tukey test). HR, heart rate; LVDP, left ventricular developed pressure.
